# Endophytic nitrogen-fixing bacteria DX120E inoculation altered the carbon and nitrogen metabolism in sugarcane

**DOI:** 10.3389/fmicb.2022.1000033

**Published:** 2022-11-07

**Authors:** Ying Qin, Xian-Qiu Xie, Qaisar Khan, Jiang-Lu Wei, An-Ni Sun, Yi-Mei Su, Dao-Jun Guo, Yang-Rui Li, Yong-Xiu Xing

**Affiliations:** ^1^College of Agriculture, Guangxi University, Nanning, China; ^2^College of Life Sciences and Engineering, Hexi University, Zhangye, China; ^3^Guangxi Key Laboratory of Sugarcane Genetic Improvement, Key Laboratory of Sugarcane Biotechnology and Genetic Improvement (Guangxi), Ministry of Agriculture and Rural Affairs, Sugarcane Research Institute of Guangxi Academy of Agricultural Sciences, Sugarcane Research Center of Chinese Academy of Agricultural Sciences, Nanning, China

**Keywords:** Biofertilizers, enzymatic activity, liquid chromatography-mass spectrometry, metabolomics, sugarcane

## Abstract

Endophytic nitrogen-fixing bacteria are versatile and widely distributed in plants. Numerous strains of endophytic nitrogen-fixing bacteria are used as biofertilizers to minimize the utilization of chemical fertilizers, improve nutrient use efficiency, increase crop productivity, and reduce environmental pollution. However, the mechanism underlying the interaction between nitrogen-fixing bacteria and plants is still unclear. So, the present study was planned to assess the effects of endophytic nitrogen-fixing bacteria on sugarcane by analyzing the changes in physiological and biochemical activities. In the current study, *Klebsiella variicola* DX120E, an endophytic nitrogen-fixing bacterium, was inoculated on sugarcane varieties B8 and ROC22 to evaluate the effects on nitrogen and carbon metabolism-related enzymatic activity and biomass. Results showed that DX120E inoculation improved the enzymatic activities related to gluconeogenesis and nitrogen metabolism increased the sugarcane plant’s height, cane juice Brix, biomass, chlorophyll, and soluble sugar content in sugarcane. Metabolomics analysis revealed that the metabolome modules were highly enriched in carbon and nitrogen metabolic pathways of strain-affected sugarcane than uninoculated control. The identified carbohydrates were associated with the glycolysis or gluconeogenesis and tricarboxylic acid (TCA) cycle in plants. Metabolomic profiling in the present investigation showed that carbohydrate metabolism is coordinated with nitrogen metabolism to provide carbon skeletons and energy to amino acid synthesis, and amino acid degradation results in several metabolites used by the citric acid cycle as an energy source. Moreover, differentially expressed metabolites of non-proteinogenic amino acids have a further complementary role to the action of endophytic nitrogen-fixing bacteria. Meanwhile, a significant difference in metabolites and metabolic pathways present in stems and leaves of B8 and ROC22 varieties was found. This study discovered the potential benefits of DX120E in sugarcane and suggested candidate regulatory elements to enhance interactions between nitrogen-fixing microbes and sugarcane.

## Introduction

China is the third-largest sugarcane (*Saccharum* spp.) producing country worldwide, and Guangxi has the largest planting area accounting for 63% of the total sugarcane cultivation in the country (Huang et al., 2020). Sugarcane is an important crop known worldwide for its high sugar and lignocellulosic biomass production, sequestering thousands of tons of atmospheric CO_2_ per hectare during its life. Many mineral fertilizers are commonly used to achieve satisfactory yield and sustainable revenue from sugarcane cultivation (Brackin et al., 2015). However, the increasing consumption of fertilizers has created negative impacts on resources by spending large amounts on imorrting chemical fertilizer which considerably has increased the agricultural budget. The microbial population has immense potential to attain agricultural sustainability in present environmental conditions.

The microbe-sugarcane system has high nitrogen (N) fixation capacity due to the availability of various biological nitrogen fixing (BNF) microorganisms, such as free-living bacteria in soil and endophytic N-fixing in plants (Xing et al., 2016). The primary reason of interaction between BNF and plants is the nutritional exchange because plants provide the carbon source required for microbial growth, and in return, microorganisms provide the fixed N to plants and promote their growth (Kirizii et al., 2007; Bulgarelli et al., 2017). It has been proven that the positive interactions between sugarcane and microorganisms in the endosphere have improved plants performance under various environmental conditions (Pitiwittayakul et al., 2021). In this alternative mechanism, the atmospheric N_2_ is reduced to ammonia catalyzed by bacteria and archaea nitrogenase enzymes. N can be absorbed by sugarcane from the soil as nitrate, ammonia, or in ammonium (NH_4_^+^) form. Nitrate reductase (NR), glutamine synthetase (GS), and glutamine oxoglutarate aminotransferase have been used to assess the influence of endophytic bacteria on the uptake of N from the soil, its metabolism and use efficiency in plants. Studies have shown that N-fixing bacteria not only promote N uptake in crops but also have an enhanced effect on photorespiration and carbohydrate metabolism (Bisht et al., 2020; Du et al., 2022). Hence, the use of BNF bacteria as a source of biofertilizer (Din et al., 2021) gives an alternative for substituting chemical fertilizers and reduces the application of N from inorganic sources in sugarcane crops (Crusciol et al., 2020).

It has been realized that endophytic N-fixing bacteria contribute to sustainable cropping system, however, the benefit efficacy differs widely, from significant to none significant in the different crops under various environmental conditions. The developments of advanced tools, techniques, and analytical methods, have identified many vital molecules involved in the signaling during the endophyte colonization in the host plants. Among several such omics technologies, metabolomics has become a popular method that has successfully analyzed several high molecular weight compounds in plants in response to microbes during the physiological, biochemical, and metabolic studies (Jayakumar et al., 2020). In addition to regulating specific metabolites within the endosphere, the endophyte microbiome also biotransforms plant-produced metabolites and subsequently stimulates the production of other compounds (Pang et al., 2021). However, still, little is known about the biochemical processes underlying plant-microbe’s interactions or the immediate metabolic alterations occurring in plants and microbes on interaction. Previously, we isolated and characterized *Klebsiella variicola* DX120E from sugarcane and demonstrated its N-fixing activity in gnotobiotic culture (Lin et al., 2015). The species was highly colonized in roots and stimulated the growth of sugarcane plantlets (Wei et al., 2014). However, the effects of fertilizer levels on sugarcane metabolites and their relationship are still unclear. To further evaluate the impact of potential N-fixing strain DX120E on sugarcane photosynthetic capacity, sucrose and N metabolism the current study was designed. To achieve the objectives of the study to disclose chemical mechanism of bacterial strain and sugarcane interaction and its effects on commercial traits of commercial sugarcane, an advanced metabolomics investigation was carried out by liquid chromatography-mass spectrometry (LC–MS) after inoculation of bacterial strain on sugarcane plants.

Sugarcane varieties ROC22 and RB86-7515 (B8) used in this study were introduced from Taiwan (China) and Brazil, respectively. It was found that DX120E affect carbohydrates and amino acids metabolism in sugarcane and promotes growth. It suggests that the interaction is based on consistent physiological and biochemical mechanisms which ensure the mutual nutritional interest of plants and bacteria. So, DX120E bacteria can be used as a potential alternative for N to achieve the goal of sustainable agriculture.

## Materials and methods

### Preparation of bacterial suspensions

*Klebsiella variicola* DX120E preserved in Guangxi Key Laboratory of Sugarcane Biology at Guangxi University (Lin et al., 2015) was gradient diluted and inoculated on Luria-Bertani media to culture without antibiotics. Single colonies were picked from liquid medium containing antibiotics (Gm15) for expansion. The liquid bacterial was centrifuged at 4000 rpm (4°C) for 10 min and suspended in sterile phosphate buffer pH 7.4; OD_600_ ≈ nm and the bacterial solution concentration was about 10^8^ CFU/ml.

### Pot experiments

The experiments from 2020/4 to 2020/11 were conducted in the greenhouse of College of Agriculture, Guangxi university in Nanning City (22° 51’ N 108° 17′ E). Sugarcane varieties ROC22 and B8 were used as the plant materials. After soaking for 1 h in the strain suspension solution, single bud setts were subjected to germination for 4 days at 28°C. The buds with visible roots were planted in sterilized sand and transplanted into pots filled with sterilized soil when the plants grew to have 3–4 leaves. Plants were watered with DX120E strain suspension twice, on the first and seventh day, after moving into pots and sterile phosphate buffer was used for the control. The pots were 30 cm in height and 24 cm in caliber. There was no fertilizer application during the growth except for normal watering and weeding. After inoculation, the agronomic traits and physiological indexes were measured at 30th, 60th, and 90th days. The properties of soil were as follows: pH 6.8, N 1.65 g/kg, phosphorus (P) 0.88 g/kg, potassium (K) 18 g/kg, and nitrate N 1.58 mg/ kg, ammonia N 2.7 mg/kg, hydrolyzed N 109 mg/kg, available P 22.0 mg/kg, inorganic P 223 mg/kg, quick-acting K 183 mg/kg, slow-acting K 232 mg/kg.

### Agronomic traits, and physiological and biochemical indexes

Plant height, stem diameter, chlorophyll content, soluble sugar, NR (Wei et al., 2007), GS (Li et al., 2002), sucrose synthase (SuSy), sucrose phosphate synthase (SPS)(Datir and Joshi, 2016) activities were determined at 30th, 60th, and 90th days after treatment and biomass and Brix were detected at the maturity stage of sugarcane plants.

### Sample collection for sugarcane metabolome analysis

The first fully expanded leaf (leaf +1) and internode 13 from bottom to top were collected from the sugarcane plants. Each treatment had six replicates and sample code information has been shown in Table 1. The leaf and internode samples were cut into small pieces, and 2–3 g of each sample was packed in a sterile centrifuge tube separately, quickly frozen in liquid nitrogen and stored in a − 80°C freezer for metabolomics analysis.

**Table 1:** The information of samples.

Sugarcane variety	Sampling position	Strains	Code
B8	Stem	CK	S-BCK
		DX	S-BDX
	Leaf	CK	L-BCK
		DX	L-BDX
ROC22	Stem	CK	S-RCK
		DX	S-RDX
	Leaf	CK	L-RCK
		DX	L-RDX

### Metabolite extraction

The chloroform-methanol method with some modifications, was applied to extract metabolites from the collected samples (De Vos et al., 2007). A total of 200 mg (±1%) of the sample were weighed in a 2 ml EP tube, and 0.6 ml 2-chlorophenyl alanine (1.34 μg/ml) methanol (−20) (±1%) was added and vortexed for 30 s. Then, the samples with 100 mg glass beads were ground for 90 s at 60 Hz in a high-throughput tissue grinder and centrifuged at 12000 rpm for 10 min (25°C). After an ultrasound performance at room temperature for 15 min, 300 μl of the supernatant was filtered through a 0.22 μm membrane, and the filtrate was added to the detection bottle of the LC–MS equipment.

### LC–MS analysis

Chromatographic separation was accomplished in a Thermo Vanquish system equipped with an ACQUITY UPLC® HSS T3 (150 × 2.1 mm, 1.8 μm, Waters) column maintained at 40°C and the temperature of the autosampler was set at 8°C. The mobile phase A is acetonitrile and B is ultrapure water. Gradient elution of the analytes was carried out with 0.1% formic acid in water (B1) and 0.1% formic acid in acetonitrile (A1) or 5 mM ammonium formate in water (B3) and acetonitrile (A3) at a flow rate of 0.25 ml/min. Injection of 2 μl of each sample was done after equilibration. The positive ion mode and the negative ion mode use different eluents, A1-B1 for positive ions and A3-B3 for negative ions. Sample measurements were performed with a gradient program: 0–1 min, Positive ions: 2% A1 + 98% B1, Negative ions: 2% A3 + 98% B3. 1–9 min, Positive ions: A1: 2% up to 50%, B1: 98% down to 50%; Negative ions: A3: 2% up to 50%, B3: 98% down to 50%. 9–12 min, Positive ions: A1: 50% up to 98%, B1: 50% down to 2%; negative ions: A3: 50% up to 98%, B3: 50% down to 2%. 12–13.5 min, positive ions: 98% A1 + 2% B1; negative ions: 98% A3 + 2% B3. 13.5–14 min, positive ions: A1: 98% down to 2%, B1: 2% up to 98%; negative ions: A3: 98% down to 2%, B3. 2 to 98%. 14–20 min, positive ions: 2%A1 + 98% B1 and 14–17 min, negative ions 2% A3 + 98% B3. The effluent was alternatively connected to an Thermo Q Exactive Plus mass spectrometer for the ESI-MSn experiments.

The ESI-MSn experiments were performed on a Thermo Q Exactive Plus mass spectrometer with the spray voltage of 3.5 kV and − 2.5 kV in positive and negative modes. Sheath gas and auxiliary gas were set at 30 and 10 arbitrary units, respectively. The capillary temperature was 325°C. The analyzer scanned over a mass range of m/z 81-1 000 for full scan at a mass resolution of 70 000. Data dependent acquisition (DDA) MS/MS experiments were performed with HCD scan. The normalized collision energy was 30 eV, and dynamic exclusion was implemented to remove some unnecessary data in MS/MS spectra (Want et al., 2010).

### Statistical and data analysis

The acquired mass spectra were exported to data format (mzXML) files of Proteowizard Software (version v3.0.8789) (Smith et al., 2006). Data pre-treatment procedures, such as peak identification, filtration, and alignment, were performed in the XCMS package (Rv3.3.2). The main parameters set were BW = 2, ppm = 15, peak width = c (5, 30), mzwid = 0.015, MZDIFF = 0.01, method = center wave. Detailed parameters for the data analyses were provided in the online appendix. Open database sources including Metlin^1^, MoNA,^2^ KEGG(Kyoto Encyclopedia of Genes and Genomes), and Metabo Analyst^3^ were used to identify metabolic pathways.

Autoscaling, mean-centering, and scaling to unit variance were used to get a reliable and intuitive data before multivariate statistical analysis. The multivariate statistical analyses in this analysis included principal component analysis and partial least squares-discriminant analysis (PLS-DA) by the Ropls package of R language (Thévenot et al., 2015). In addition, the student t-test was also applied to measure the significance of data analysis. Statistical analyses were performed using SPSS software version 23.0 (IBM Corp, Armonk, New York). An adjusted value of *p*<0.05 was considered statistically significant. The resulted graph of the data was produced in Origin (2016) software.

## Results

### Enzyme activity related to carbon and N metabolisms

**Figure 1:**
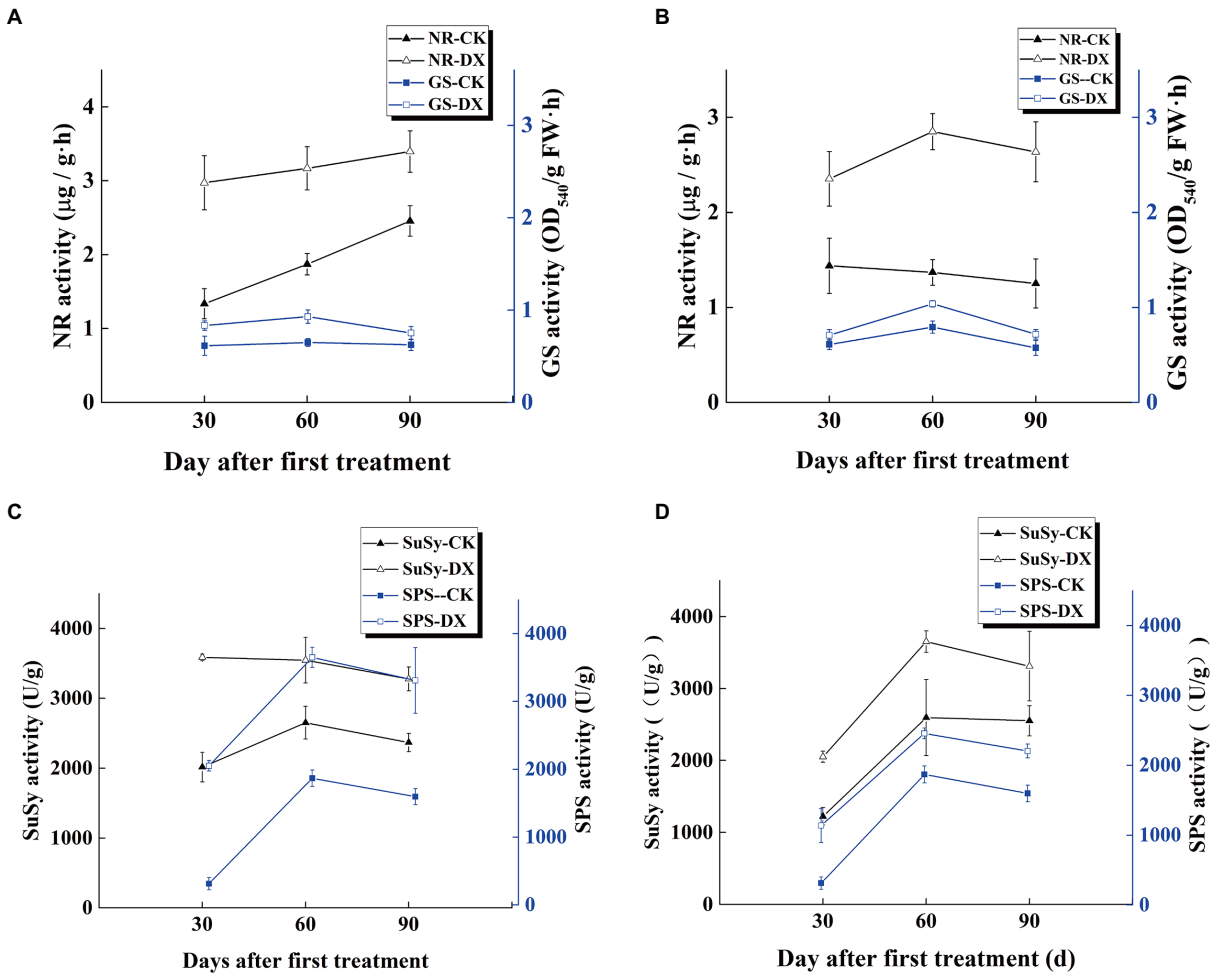
Trends of nitrogen and carbon metabolisms-related enzymes activities in the leaves of two sugarcane varieties inoculated with *Klebsiella variicola* DX120E compared with control. **(A)** Nitrate reductase (NR) and Glutamine synthetase (GS) activities in sugarcane variety B8; **(B)** NR and GS activities in sugarcane variety ROC22; **(C)** sucrose synthase (SuSy) and sucrose phosphate synthase (SPS) activities in B8; **(D)** SuSy and SPS activities in ROC22; CK, without microbial inoculation; DX, inoculation with DX120E. Data are the means of three replicates. Error bars represent the standard deviation.

Under the nitrogenous condition, the effects of DX120E on the activity of NR and GS in leaves were different in both sugarcane varieties at different stages (Figures 1A,B). Inoculation of strain increased the NR and GS activity in B8 and ROC22 at three-stage, especially at 60th day. The NR activity was increased in B8 from the young to mature stages but decreased in ROC22 at the mature stage. The GS activity was increased at first and then decreased at the mature stage of both sugarcane varieties.

The results indicated that the activities of SPS and SuSy in leaves of sugarcane had significant differences in response to DX120E inoculation in sugarcane plants. SuSy and SPS presented first rise and then fall trend in both sugarcane varieties, with the highest activity at 60th day of treatment. The effects of DX120E strain inoculation treatment on the enzyme activity were more significant in variety B8 than ROC22 on the 30th and 90th day. The SuSy activity in the DX120E strain inoculation treatment increased 1.78 and 1.68-fold at the 30th day, and 1.38 and 1.30-fold at the 90th day in B8 and ROC22 varieties compared with the control, respectively. The SPS activity was increased by 6.56 and 3.64-fold in B8 and ROC22 varieties at the 30th day and 2.07 and 1.38-fold at the 90th day, respectively (Figures 1C,D).

### Quantitative and qualitative traits

**Figure 2:**
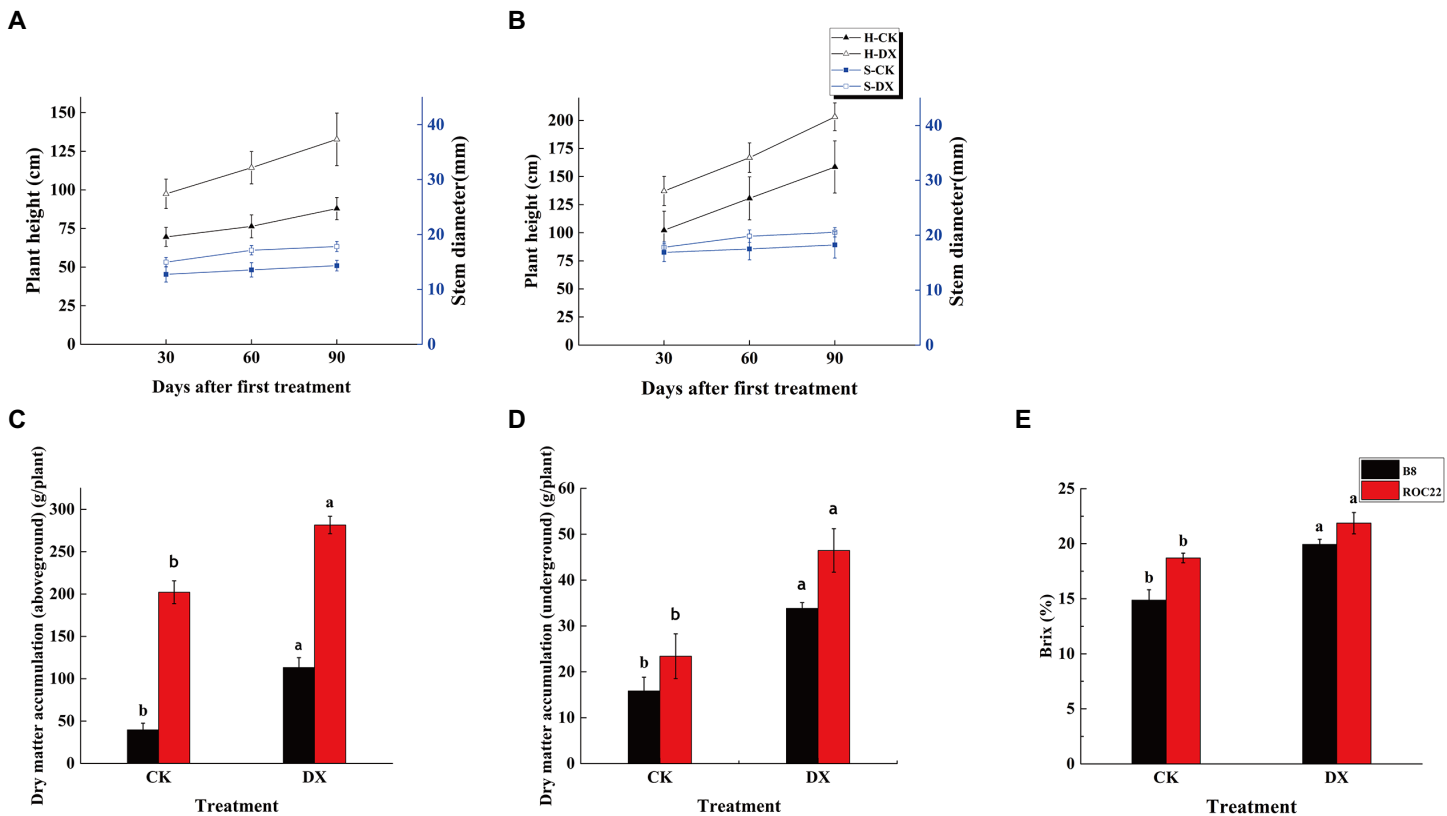
Performances of the agronomic traits in two sugarcane varieties inoculated with *Klebsiella variicola* DX120E compared with control. **(A)** Plant height and stem diameter in sugarcane variety B8; **(B)** Plant height and stem diameter in sugarcane variety ROC22; **(C)** Aboveground dry matter weight in sugarcane variety B8 and ROC22; **(D)** Underground dry matter weight in sugarcane variety B8 and ROC22; **(E)** Brix in the stem at maturity stage. CK, control without microbial inoculation. DX, inoculation with DX120E. Data were the means of three replicates. Error bars represent the standard deviation. Different lowercase letters on the column show the statistical significance at *p* ≤ 0.05.

The sugarcane varieties B8 and ROC22 responded positively to the inoculation with DX120E by increasing the plant height, stem diameter, biomass, and Brix (Figure 2). At the 30th, 60th, and 90th day after DX120E inoculation, the plant height in the varieties B8 and ROC22 was increased by 1.4, 1.5, 1.71, and 1.34, 1.28, 1.28 times, respectively than the control (Figures 2A,B). For stem diameter, the effects of bacterial inoculation were more evident in the variety B8 than ROC22, and the increase was 1.71 times higher in S-B-DX than S-B-CK on the 90th day (Figures 2A,B). Similarly, the above and underground dry matter accumulation showed a positive response to inoculation through increasing the aboveground part by 1.45 times and the underground part by 2.44 times (Figures 2C,D). The Brix was increased in B8 and ROC22 treatment plants by 34 and 16.95%, respectively, than the control (Figure 2E).

### Chlorophyll and soluble sugar contents

**Figure 3:**
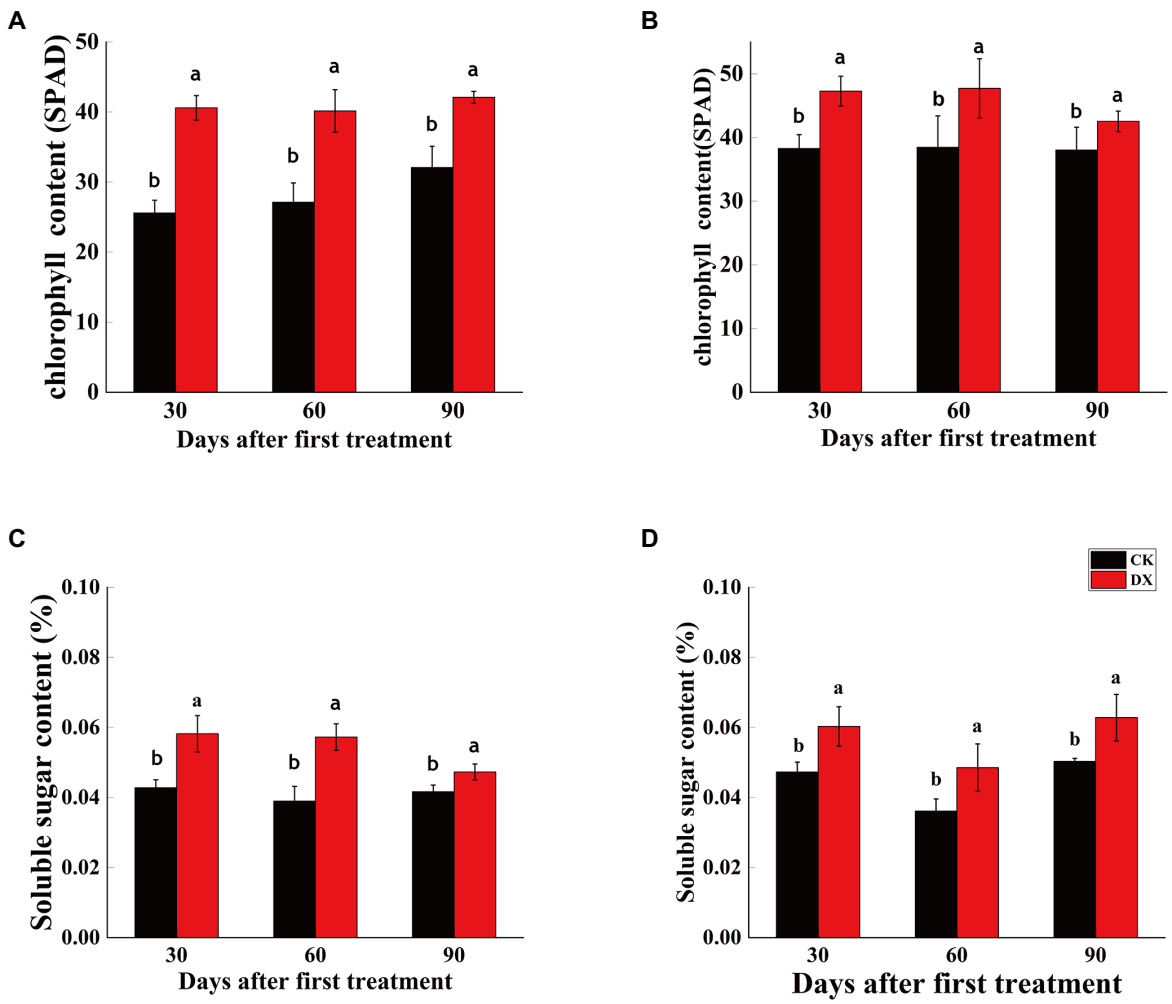
Chlorophyll content (**A,B**) and soluble sugar content (**C,D**) in leaves of two sugarcane varieties inoculated with *Klebsiella variicola* DX120E compared with control. **(A)** Chlorophyll content in sugarcane variety B8; **(B)** Chlorophyll content in sugarcane variety ROC22; **(C)** soluble sugar content in sugarcane variety B8; **(D)** soluble sugar content in sugarcane variety ROC22; CK, control without microbial inoculation; DX, inoculation with DX120E. Data are means of three replicates. Error bars represent the standard deviation. The lowercase letters on the column show the statistical significance at *p* ≤ 0.05.

Both chlorophyll content and soluble sugar content were significantly higher in the leaves of sugarcane plants inoculated with DX120E than control. However, the chlorophyll content gradually decreased and soluble sugar content fluctuated in sugarcane variety ROC22 (Figures 3B,D). Soluble sugar content gradually decreased and chlorophyll content remained unchanged in sugarcane variety B8 (Figures 3A,C).

### Multivariate statistical analysis

**Figure 4:**
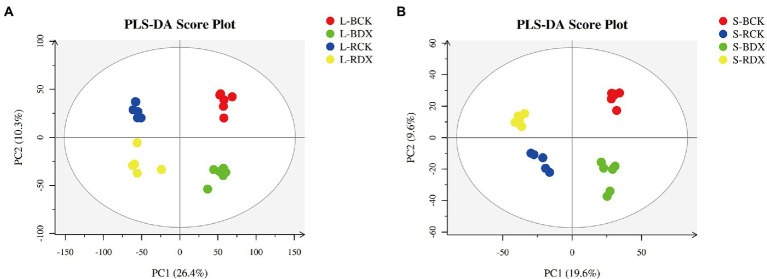
Score plots of principal component analysis for 8 sugarcane samples. **(A)** Leaf samples; **(B)** stem samples; L-BCK, the leaf sample of sugarcane variety B8 in control; L-BDX, the leaf sample of B8 in the treatment with *Klebsiella variicola* DX120E; L-RCK, the leaf sample of sugarcane variety ROC22 in control; L-RDX, the leaf sample of ROC22 in the treatment with *Klebsiella variicola* DX120E; L, leaf; S, stem.

The leaves and stems of sugarcane varieties inoculated with DX120E and control were sampled for LC–MS analysis. PLS-DA of metabolites analysis was performed to get the general functional groups in response to the treatments. Analysis indicated that the first principal component (PC1) separated the two sugarcane varieties and explained 26.4 and 10.3% of the total variation in stem and leaf, respectively (Figure 4A). The second principal component (PC2) separated the control and treatment samples explaining 19.6 and 9.6% of the total change, indicating that the level of metabolites in the leaf and stem was significantly different (Figure 4B). The parameters presented stability and predictability of the model and effectively showed the significant distinctions in the metabolic profiles between the metabolites of different sugarcane varieties between the DX120E inoculation treatment and the control.

### Impact of DX120E on sugarcane metabolome

**Figure 5:**
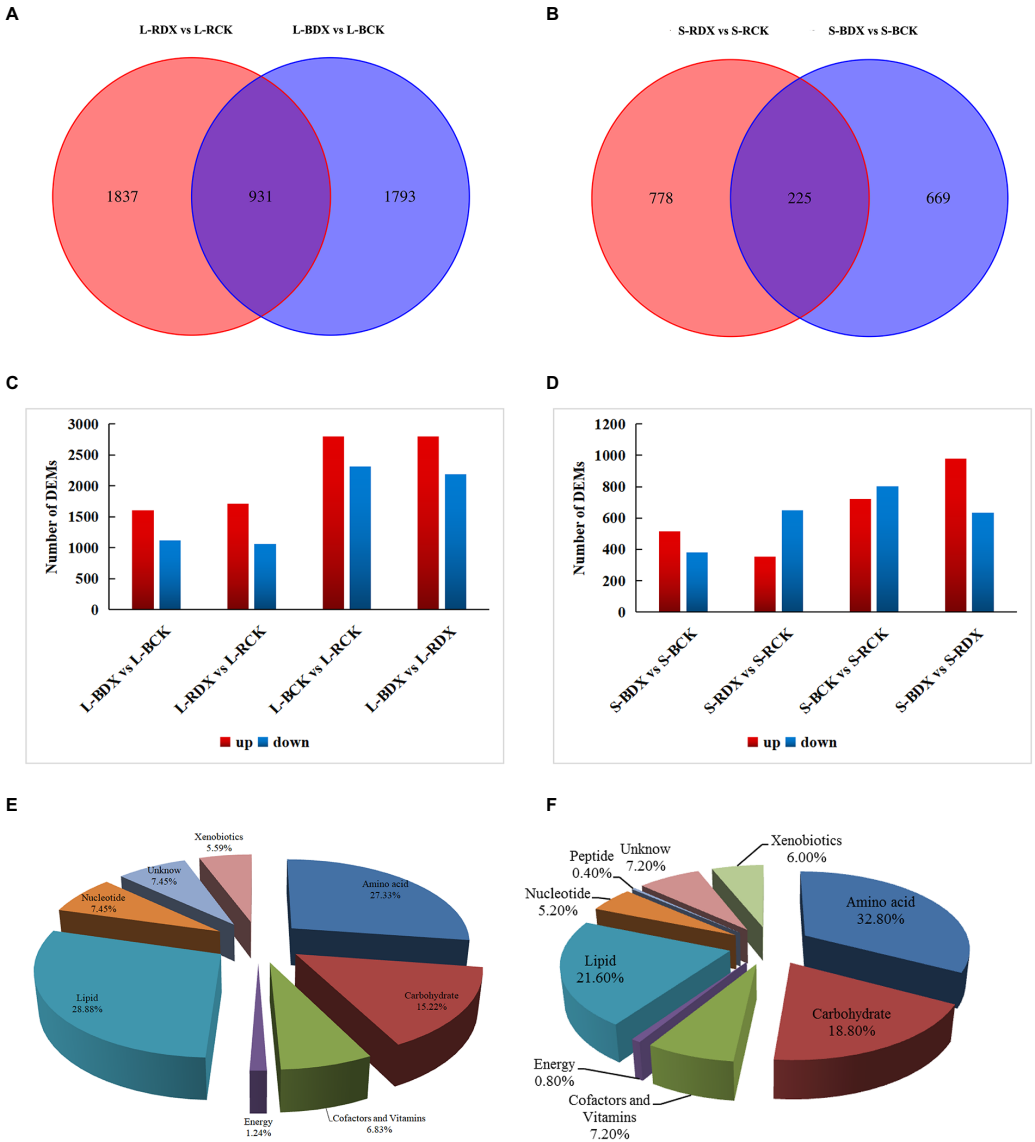
Differentially expressed metabolites (DEMs) detected from leaves and stems of two sugarcane varieties inoculated with *Klebsiella variicola* DX120E compared with control. Venn diagrams show the overlapping and interconnection between DEMs in leaf **(A)** and stem **(B)** at different treatments, column charts indicate the numbers of DEMs in different groups of the leaf **(C)** and stem **(D)**, and pie charts **(E,F)** display all the metabolites identified in leaf and stem, respectively.

Screening conditions for differential metabolites were based on VIP > 1 and *p* < 0.05. Fold change>1 in differential substances is upregulated and Fold change<1 is downregulated. A total of 4,561 differentially expressed metabolites (DEMs) were identified and relatively quantified in sugarcane leaf at p<0.05. A comparison between L-BDX vs. L-BCK found 2,724 DEMs, of which 1,606 were up-regulated and 1,118 down-regulated. In the L-RDX *VS* L-RCK, 2,768 DEMs were screened, of which 1710 (61.78%) were up-regulated and 1,058 (38.22%) down-regulated (Figures 5A,C). From L-BDX vs. L-BCK and L-RDX vs. L-RCK groups, only 931 shared DEMs were detected. Venn analysis identified 894 DEMs in stem of B8, among them 515 (57.61%) were up-regulated, and 379 (42.39%) down-regulated. A sum of 1,003 DEMs were obtained from the stem samples of ROC22, of which 353 (35.09%) were up-regulated, and 650 (64.61%) down-regulated (Figures 5B,D). The total number of DEMs was slightly higher in ROC22 (3771) than in B8 (3618). To further understand the functions and related biological processes of the DEMs, they were blasted in KEGG and Metabolon Inc. databases for functional annotation. The results showed 322 various substances were obtained in the leaf and 250 in the stem sample. According to their nature and functions, these substances were classified into carbohydrate, energy, lipid, nucleotide, amino acid, xenobiotics, peptides, cofactors, and vitamins. The classification showed that 28.88% of the DEMs in leaf were associated with lipids, 27.33% with amino acids, and 15.22% with carbohydrates; 32.8% of the DEMs in stem were linked with amino acids and 18.8% with carbohydrates, constituting more than half of the total DEMs (Figures 5E,F). Significantly enriched metabolites were identified in “carbohydrate metabolism” and “amino acid metabolism.” This is direct evidence that the DX120E inoculation significantly influenced the carbon and amino acid metabolism in sugarcane.

### Effect of DX120E on carbon and amino acid metabolites in sugarcane

**Figure 6:**
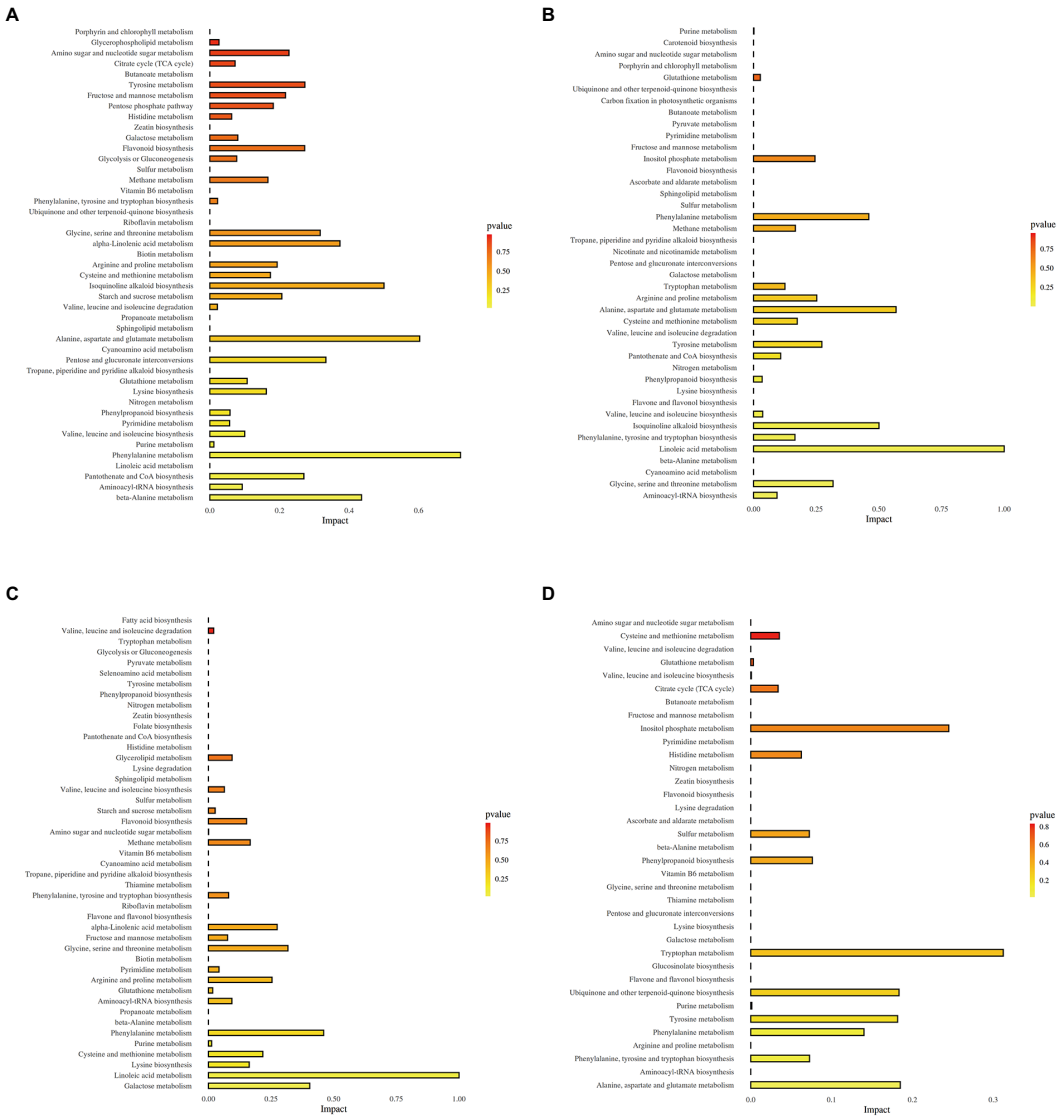
Metabolic pathways detected from leaves and stems of two sugarcane varieties inoculated with *Klebsiella variicola* DX120E compared with control. **(A)** L-BDX vs. L-BCK; **(B)** S-BDX vs. S-BCK; **(C)** L-RDX vs. L-RCK; **(D)** S-RDX vs. S-RCK.

The KEGG enrichment analysis of DEMs was conducted to understand the specific metabolic pathways in the leaves and stems of two sugarcane varieties. 115 DEMs of L-BDX vs. L-BCK group and 101 DEMs of L-RDX vs. L-RCK group were enriched in 45 metabolic pathways (Figures 6A,C). 64 different metabolites and 41 metabolic pathways were changed in stem of B8 after DX120E inoculation compared with the control (Figure 6B). Meanwhile, 53 different metabolites and 42 metabolic pathways were identified in S-RDX vs. S-RCK (Figure 6D). In total, 59 metabolic pathways were obtained from the leaf and stem samples, and 14 of them were related to carbon metabolism and 19 associated with amino acid metabolism, accounting for 55.93% of the total enriched metabolic pathways.

### Effects of DX120E inoculation on carbon metabolism

**Figure 7:**
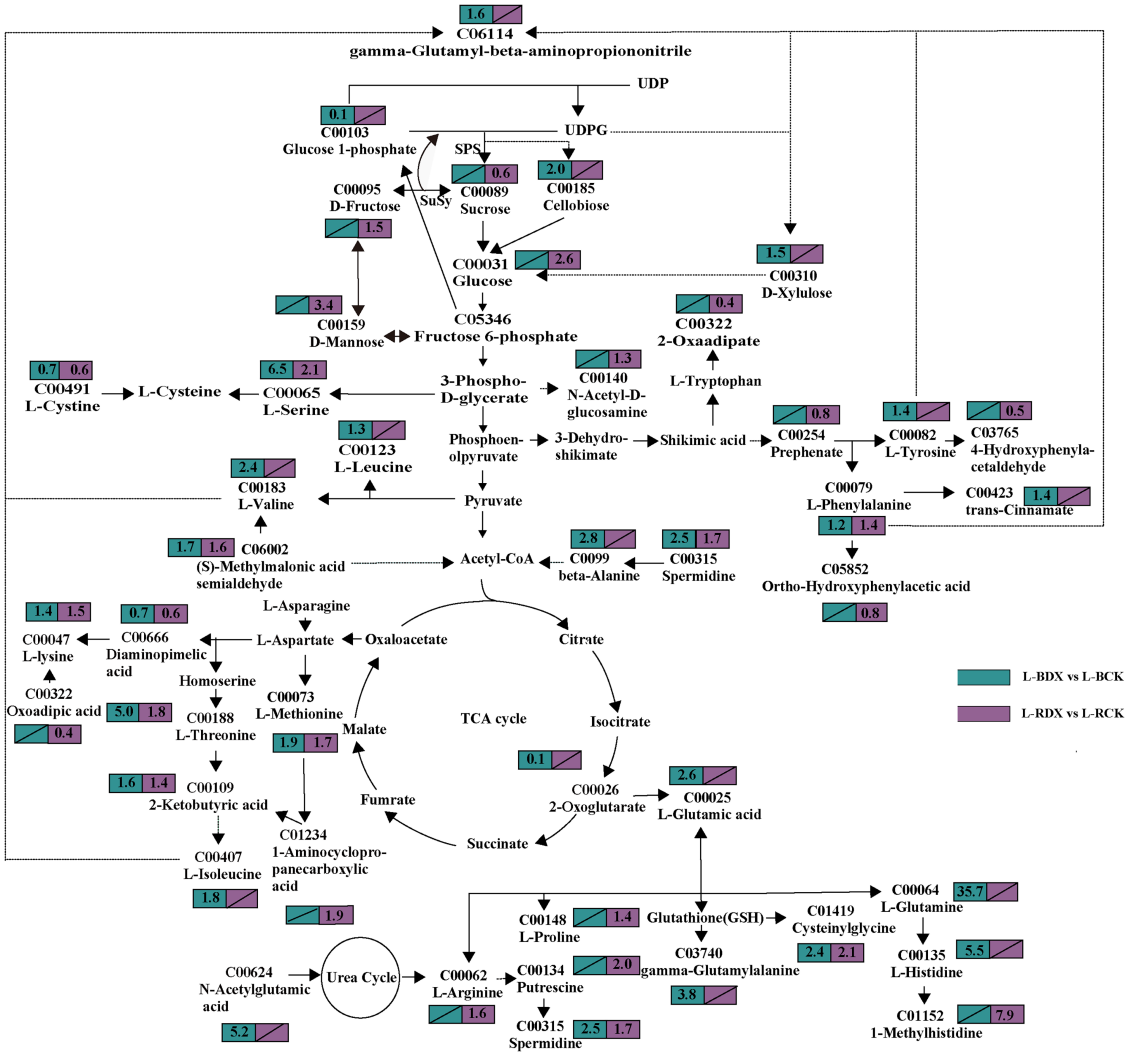
Effects of *Klebsiella variicola* DX120E inoculation on the carbon and amino acid metabolism in sugarcane leaf compared with control. The number in the rectangle represents the fold change of different metabolites in different combinations, and the slash represents no significant difference.

**Figure 8:**
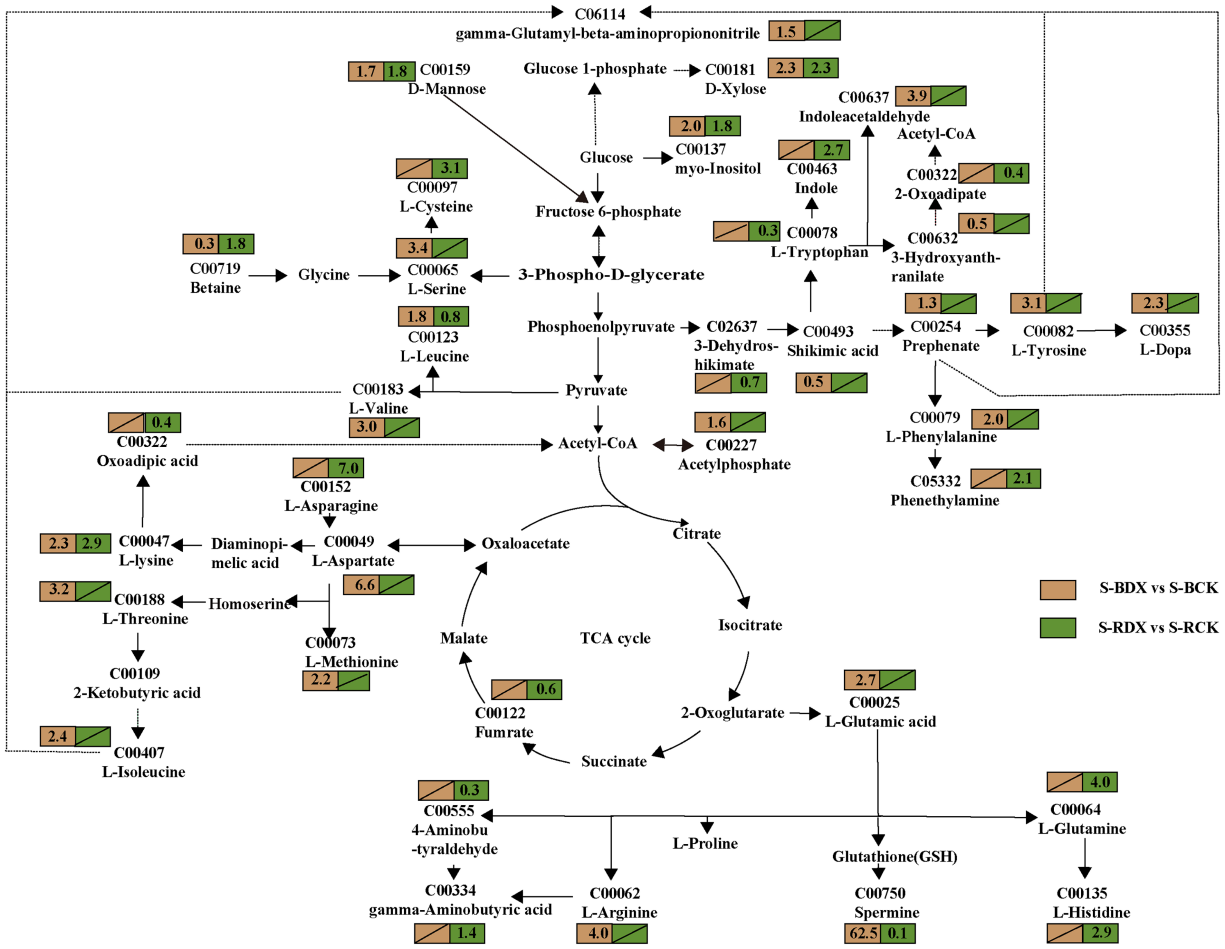
Effects of *Klebsiella variicola* DX120E inoculation on the carbon and amino acid metabolism in stem of sugarcane as compared with control. The number in the rectangle represents the fold change of different metabolites in different combinations, and the slash represents no significant difference.

In order to identify the metabolites involved in sugarcane carbon metabolism, we further analyzed the expression of the DEMs in the metabolic pathways closely related to carbon metabolism. Thirty-seven DEMs related to carbohydrates were obtained from the leaves and stems (Supplementary Table S1). The mapped carbohydrate metabolite D-mannose (C00159) showed an upward trend in both leaf and stem. Cellobiose (2.0-fold), sucrose (0.6-fold), D-glucose (2.6-fold), D-fructose (1.5-fold), glucose 1-phosphate (0.1-fold), fructose 6-phosphate (0.4-fold), N-acetyl-D-glucosamine (1.3-fold), D-xylulose (1.5-fold), D-xylulose (1.6 and 1.4-fold) showed high fluctuations in leaf only (Figure 7). The expressions of myo inositol, D-xylose, and acetyl phosphate showed up-regulation, but fumaric acid was down-regulated in stem (Figure 8). The data in Figure 7 suggested that induction of the metabolites participating in carbon metabolism may be regulated by fructose and mannose metabolism, glucosinolate biosynthesis, amino sugar and nucleotide sugar metabolism, ascorbate and aldarate metabolism, butanoate metabolism, citrate cycle, carbon fixation, glycolysis or gluconeogenesis, inositol phosphate metabolism, pentose and glucuronate interconversions, pentose phosphate pathway, propanoate metabolism, pyruvate metabolism, and starch and sucrose metabolism.

### Effects of DX120E inoculation on amino acid metabolism

Carbohydrate and amino acid metabolisms are closely linked with glycolysis and the tricarboxylic acid (TCA) cycle. One-hundred fourteen amino acid-associated metabolites were obtained from leaves and stems of sugarcane (Supplementary Table S2). Among them, 38 were identified in leaves and 28 in stems of B8 (B-DX vs. B-CK). Twenty-nine metabolites were found in leaves and 19 in stems of ROC22 (R-DX vs. R-CK). The families of ubiquitous amino acids such as glutamate (Glu) family (C00025, C00064, C00062, C00148), aspartate (Asp) family (C00073, C00188, C00407, C00047), pyruvate family (C00183, C00123), sering (Ser) family (C00065, C00491), aromatic amino acid family (C00079, C00082), and histidine (His) (C00135) were observed in leaf under the DX120E inoculation conditions (Figure 7). It was observed that 12 amino acids were up-regulated in S-BDX, but only 5 were up-regulated and 2 down-regulated in response to DX120E strain in the stem of ROC22 compared with control (Figure 8). The differentially expressed free amino acid shown in leaves was L-proline, and those shown in the stems were L-aspartic acid and L-tryptophan. In addition to the free amino acids, non-proteogenic amino acids such as β- alanine varied in the tissue of sugarcane inoculated with DX120E. These results demonstrated that the strain DX120E inoculation altered the carbon metabolism and induced amino acid metabolism.

## Discussion

Studies have shown that inoculation with associative plant-growth-promoting bacteria increased sugarcane plant growth and productivity (Singh et al., 2020). The benefits of the interactions between endophytic diazotrophic bacteria and plants in the family Poaceae, such as sugarcane (Carneiro et al., 2021), maize (Sheoran et al., 2021), wheat (Rana et al., 2020), and rice (Khan et al., 2020) have already been reported. The results of this study confirmed the beneficial effects of DX120E inoculation on sugarcane’s growth and Physio-Biochemical characteristics. Sugarcane plants inoculated with DX120E showed increased plant height, stem diameter, biomass, and Brix (Figure 2) compared with the control, which was similar to the results in previous studies (Kifle and Laing, 2016). It has been reported that the rhizosphere bacteria could induce the production of soluble phosphate, indole-3-acetic acid phytohormone, and siderophores secretion in sugarcane, which positively affected the root architecture, root length, plant height, and stem diameter (Bononi et al., 2020; Abiraami et al., 2021). However, little is known about fluctuations and regulatory mechanisms of metabolites in endophytic bacteria-plant interaction systems. Endophytic microorganisms can promote the growth of a plant and accelerate production of primary and secondary metabolite accumulation.

Carbon and N metabolism are the two essential pathways in plant growth and product formation. The role of endophytic N-fixing bacteria in improving carbon and sugar metabolisms, photosynthetic carbon assimilation, starch and sucrose metabolism, carbohydrate transport and utilization process in plants has been proven (Coruzzi and Zhou, 2001; Zheng, 2009). The sucrose formed by photosynthesis in sugarcane leaves is transported to the phloem, loaded to the sieve tube for long-distance transport, and finally unloaded through the phloem into the parenchyma cells in the stem for storage (Khan et al., 2021; Okamoto et al., 2021). During the transportation process, part of the sucrose is hydrolyzed to fructose and glucose in the growing part for plant growth. The present study found that the activities of SPS and SuSy in the plants inoculated with DX120E were higher than those in control during the growth cycle (Figures 1C,D). SPS mainly catalyzes the combination of uridine diphosphate glucose and fructose-6-phosphoric acid to form sucrose phosphate, while SuSy is mainly responsible for catalyzing the decomposition of sucrose in the process of sucrose synthesis and metabolism (Wang et al., 2019; Khan et al., 2022). In addition, metabolomics analysis results showed that glucose-1-phosphoric acid and uridine diphosphate glucose form cellobiose, the product of cellulose hydrolysis. Cellulose is an essential component of the plant cell wall and the primary sensing mechanism of biological and abiotic signals and plays a vital role in growth, development, cell shape and defense by regulating cell volume and turgor (Zhang and Zhang, 2020; Ballesteros et al., 2021). These may be the reason that glucose, D-fructose, and cellobiose were detected in sugarcane. The relative contents of D-mannose and D-xylulose were also elevated in the stem after DX120E inoculation (Figures 7, 8). Xylulose formed by the glucuronic acid pathway can be connected to the pentose phosphate pathway. In addition to generating energy, the pentose phosphate pathway mainly provides a variety of raw materials for anabolism, such as NADPH for the biosynthesis of fatty acids and cholesterol, ribose 5-phosphate for the synthesis of nucleotide coenzymes and nucleotides, and erythritose 4-phosphate for the synthesis of aromatic amino acids (Sharkey, 2021). It is considered that the application of PGPB strains can positively induce improvement of sucrose storage, photosynthesis rate, sugar transporters activity, and carbon distribution in plants (Kohli et al., 2020; Mishra et al., 2021).

In the current study, the increased promoting influences of NR and GS after bacterial inoculation were observed in the leaves of sugarcane varieties B8 and ROC22 compared with the control (Figures 1A,B), reflecting the effects of N-fixing bacteria on the N metabolism-related enzyme activities in sugarcane. Amino acid is an integral part of N metabolism and functions as the building block of proteins and precursors for secondary metabolism (Zaynab et al., 2018). Firstly, carbohydrate metabolism provides sufficient energy and substrate for amino acid synthesis and nitrogen metabolism (Bassi et al., 2018; de Abreu et al., 2021). In this study, the contents of fructose-6-phosphoric acid (glycolysis or gluconeogenesis), 2-oxoglutarate (TCA cycle), and 2-ketobutyric acid (propanoate metabolism) decreased in leaves (Figure 7), while the content of fumarate (TCA cycle) decreased in stems after DX120E inoculation in sugarcane (Figure 8). The ubiquitous amino acid contents of 15 DEMs increased in leaves, and 17 amino acids had significant differences in stems (Supplementary Table S2), indicating that endophytic diazotroph was closely linked to carbohydrate metabolism, biological N fixation, and amino acid metabolism. Enhanced amino concentrations have also been documented by metabolomics analysis in the microbial-sugarcane system (Aguiar et al., 2018).

This study deeply proved that the differential amino acids in stems and leaves of sugarcane covered 5 families derived from Glu, Ser, pyruvate, Asp., and chorismate. The Asp family pathway in plants is crucial from the nutritional standpoint because it leads to the synthesis of four essential amino acids lysine, threonine, methionine, and isoleucine. The Asp-derived amino acids are made from oxaloacetate (citric acid cycle), while 2-oxoglutarate is the initial metabolite for the synthesis of Glu, glutamine (Gln), proline, and arginine (Galili, 2011). Asparagine and Gln act as amide and amino N sources for amino acid interconversions and function as efficient N storage and partitioning compounds (Oddy et al., 2021). The difference of L-Gln in the stem was the most obvious, with a difference of 35.71 times compared with the control. Glu and Gln are intermediaries in introducing ammonia into amino acid molecules. Plants and microorganisms form Glu by the reductive amination of ɑ-ketoglutarate through the action of Glu dehydrogenase, which provides amino groups to form many other amino acids through transamination. N is stored in arginine form in plants, which is later transformed into amino acid and urea. Arginine concentration in the plants inoculated with endophytic bacterium strain DX120E was comparatively higher and might contribute to arginine catabolism’s enhanced nitrogen N content (Figure 7). Pyruvate is the substrate for the branched-chain amino acid (valine and leucine) pathway and alanine synthesis; glycine, cysteine, and Ser are made from 3-phosphoglycerate (also from Glycolysis) (Pratelli and Pilot, 2014; Ros et al., 2014). Phosphoribosyl pyrophosphate and erythrose-4-phosphate from the pentose phosphate pathway are the substrates for His and aromatic amino acid biosyntheses. His plays important biochemical roles as a nucleophile in phosphoryl group transfer and as a metal-binding ligand (Mozafari et al., 2016). The three aromatic amino acids, tryptophan, phenylalanine, and tyrosine, are synthesized from a frequent precursor, chorismate, originating from the shikimate pathway (Wakasa and Ishihara, 2009; Adams et al., 2019). Aromatic amino acids synthesize proteins in plants, and serve as precursors of numerous natural products, such as pigments, alkaloids, hormones, and cell wall components (Maeda and Dudareva, 2012; Soares et al., 2014; Schenck and Maeda, 2018). Indole, the precursor of indole-3-acetic acid is biosynthesized from tryptophan, and L-3,4-dihydroxyphenylalanine (Soares et al., 2014) has been observed to be enhanced in sugarcane. The significance of this pathway in plants is indicated by using about 20% of the carbon is constant through photosynthesis. These statistics strongly advocate that changing the shikimate and its derivative (or downstream) pathways can lead to dramatic modifications in secondary metabolism.

In addition to 20 ubiquitous amino acids used in protein synthesis, plants synthesize over 250 non-proteinogenic amino acids concerned with synthesizing compounds that are anti-herbivory, anti-microbial and respond to N storage, abiotic stresses, and plant hormones. Dimkić et al. (2022) and Belimov et al. (2015) reported that the expression of the non-proteinogenic amino acids was related to increasing stress or associated with micro-plant interactions. One such non-proteinogenic acid is β-alanine (L-BDX vs. L-BCK), which was changed in sugarcane leaf after inoculation of DX120E in this study. It was reported that β-alanine has essential roles in plant physiology and metabolism, directly as a defense compound that enables plants to withstand various stresses such as hypoxia, waterlogging, and drought, and indirectly as a precursor to the compounds pantothenate and CoA (Parthasarathy et al., 2019), so probably improves the stress resistance of sugarcane. Therefore, it is likely that the measured non-proteinogenic amino acids played a role in the effects of bacteria on sugarcane plants. The thin line between sugarcane and endophytic bacteria needs further research.

A comparison of the functions of the identified metabolites revealed that endophytic PGPB was associated with diverse metabolic activities in sugarcane tissues, mainly carbohydrates and amino acids. The metabolic response of plants to inoculation with bacteria has been increasingly investigated in recent years, and few of those studies have analyzed the role of PGPB in shaping the tissue metabolome in carbon and N metabolism (Garcia et al., 2022; Mhlongo et al., 2022). Amino acid metabolism is closely linked to carbohydrate and ammonium N (absorbed and synthesized from nitrate) metabolisms, which are the need for protein synthesis and secondary metabolism. Amino acid biosynthesis, degradation, and energy obtained from carbohydrate metabolism led to the generation of several metabolites used as energy sources by the citric acid cycle. However, there were differences in different sugarcane genotypes and organs in response to the N-fixing bacteria. The difference in numbers and types of carbohydrates and amino acids enriched in leaves and stems of sugarcane indicated that the variety B8 was stronger than ROC22, and leaves were stronger than stems. Changes in metabolites and metabolic pathways might facilitate the bacterial penetration in the root cell wall for their host (Purushotham et al., 2020) and decrease external N requirements.

So, it was found that endophytic N-fixing bacteria has an essential role in carbon and N metabolism and improvement of sugarcane growth. The current study identified several key enzymatic activities associated with carbon and N metabolisms producing metabolites reflects the interactions between N-fixing bacteria and sugarcane. These carbohydrates, amino acids metabolites, and metabolic pathways particularly associated with non-proteinogenic amino acids could be used as markers to study the effects of endogenous N-fixing bacteria on sugarcane and understand the underlying molecular mechanisms of endophytic N-fixing bacteria interacting with plants.

## Conclusion

The current study highlighted that endophytic PGPB strain DX120E inoculation played a regulatory role in the growth promotion, physiological and metabolical activities in the host plants at different levels resulted in better performance of the agronomical traits in sugarcane compared to control. N-fixing bacteria inoculation triggered the chemical changes in sugarcane leaf and stem discovered in metabolic analysis. Most of the identified differentially expressed metabolites are involved in carbohydrates and amino acids and metabolic pathways and enriched in glycolysis and the citric acid (TCA) cycle and amino acid metabolisms. It identified that the strain DX120E inoculation influenced intricate chemical communication of carbon, N, and sugar networks in sugarcane and further improved protein synthesis and secondary metabolite production, potentially promoted plant growth and development. However, responsive differences exist between different tissues and genotypes of sugarcane. These results provide a theoretical basis for the further application of *Klebsiella* in sugarcane cultivation. Further study of individual metabolites would assist in further understanding the interaction mechanism between sugarcane and endophytic bacteria, associated bacteria, and rhizosphere microbes.

## Data availability statement

The datasets presented in this study can be found in online repositories. The names of the repository/repositories and accession number(s) can be found at: LC-MS to EMBL-EBI Metabo Lights Database: Study MTB LS 5448 (www.ebi.ac.uk/metabolights/MTBLS5448) and Study MTB LS 5417 (www.ebi.ac.uk/metabolights/MTBLS5417).

## Author contributions

YQ, X-QX, J-LW, A-NS, Y-MS, and D-JG performed the experiment and data analysis. Y-XX supervised the project and designed the study. YQ, QK, Y-RL, and Y-XX wrote the manuscript. All authors contributed to the article and approved the submitted version.

## Funding

This work was funded by the National Natural Science Foundation of China (31971858 and 31560353), Guangxi Special Fund for Scientific Base and Talent (GKAD17195100), Fund for Guangxi Key Laboratory of Sugarcane Genetic Improvement (19–185-24-K-03-01), and National Modern Agricultural Production Technology System Guangxi Sugarcane Innovation Team Project (nycytxgxcxtd-2021-03-01).

## Conflict of interest

The authors declare that the research was conducted in the absence of any commercial or financial relationships that could be construed as a potential conflict of interest.

## Publisher’s note

All claims expressed in this article are solely those of the authors and do not necessarily represent those of their affiliated organizations, or those of the publisher, the editors and the reviewers. Any product that may be evaluated in this article, or claim that may be made by its manufacturer, is not guaranteed or endorsed by the publisher.

## Footnotes

^1^ http://metlin.scripps.edu

^2^ https://mona.fiehnlab.ucdavis.edu//

^3^ www.metaboanalyst.ca
